# A multimodal and multidisciplinary program to prevent loss of mobility in patients aged over 70 years: study protocol of a multicenter cluster randomized study in primary care (the PRISME-3P study)

**DOI:** 10.1186/s12877-019-1059-5

**Published:** 2019-02-19

**Authors:** Sofia Perrotin, Thomas Gilbert, Marine Dupuis, Laurent Villeneuve, Sylvie Bin-Dorel, Amna Klich, Laurent Letrilliart, Marc Bonnefoy

**Affiliations:** 1 University Lyon, Université Claude Bernard Lyon 1, Collège universitaire de médecine générale, Lyon, France; 2 Hospices Civils de Lyon, Service de Médecine Gériatrique, Centre Hospitalier Lyon-Sud, Pierre-Bénite, France; 3 Hospices Civils de Lyon, Unité de Recherche Clinique, Pôle Information Médicale Evaluation Recherche, Lyon, France; 4 University Lyon, Université Claude Bernard Lyon 1, EMR 3738, Lyon, France; 5 University Lyon, Université Claude Bernard Lyon 1, EAM Parcours Santé Systémique, 4128 Lyon, France; 6 Hospices Civils de Lyon, Service de Biostatistique, Lyon, France; 7 CNRS UMR 5558, Laboratoire de Biométrie et Biologie Evolutive, Equipe Biostatistique-Santé, Villeurbanne, France; 8 University Lyon, Université Claude Bernard Lyon 1, Université Saint-Etienne, Collège universitaire de médecine générale, Lyon, France; 9 University Lyon, Université Claude Bernard Lyon 1, Université Saint-Étienne, HESPER EA, 7425 Lyon, Saint-Etienne France; 10 Laboratoire CarMeN (cardiovasculaire, métabolisme, diabétologie et nutrition), University Lyon, Université Claude Bernard Lyon 1, INSERM U1060, Pierre-Bénite, France

**Keywords:** Mobility, Exercise, Nutrition, Primary care, Prevention, Elderly patients

## Abstract

**Background:**

Reduced mobility is the first sign of functional decline and can lead to dependency in elderly people. Screening for the risk of mobility limitation in this population is an important public health issue to prevent further disabilities. Despite the current lack of guidelines, primary care healthcare providers may have a central role to play in this type of screening. Multi-domain physical exercise interventions in older persons have shown some efficacy/effectiveness on frailty status, yet, to the best of our knowledge, no published study has focused on patients screened in primary care.

**Method:**

The PRISME-3P study is a national, interventional, multicenter, cluster randomized trial. Patients over 70 years of age will be systematically screened by their general practitioner (GP) on the basis of clinical criteria of mobility limitation. To avoid contamination bias, the unit of randomization will be the GP practice. In the intervention group, patients will consult a geriatrician and a dietician, and will receive a physical training program from a personal trainer who will demonstrate the exercises and provide follow-up coaching. The control group will receive standard care. The primary outcome will be the change in Short Physical Performance Battery (SPPB) scores between inclusion and 6-months follow-up.

**Discussion:**

We expect an improvement of the SPPB between inclusion and 6 months of follow-up.

**Trial registration:**

This study is registered in ClinicalTrials.gov (NCT02847871, 27 July 2016).

## Background

Senior citizens are particularly exposed to the risk of dependency or functional disabilities, which represent in industrial countries a large proportion of current healthcare costs. It is also a major concern for the future considering current demographics as, although life expectancy continues to increase, life expectancy without disability tends to remain stable, and therefore the burden of disability in older people is set to increase [1, 2].

Disease, cellular aging, musculoskeletal changes, and undernutrition all contribute to decrease muscle mass which leads to sarcopenia in older adults [3, 4]. Loss of muscle mass also causes a reduced muscle strength and a decrease of maximal oxygen uptake [5]; this ultimately leads to a mobility limitation [6] and a vicious circle is established as this in turn leads to loss of muscle mass [7]. Mobility limitation is common in older adults, almost 30% in those aged over 65 years [8, 9], and the most frequently used method in the literature to measure this has been the ability to rise from a chair, walking speed, or capacity to walk 400 m [10, 11]. It is predictive of dependence and the first sign of functional decline [10, 12], which increases the likelihood of depression [13], risk of falls and fractures [14], institutionalization [15] and mortality [16], and lowers quality of life. Mobility limitation is also considered as part of the definition of frailty. For example, Fried et al. have defined frailty as a clinical syndrome in which three or more of the following criteria are present: unintentional weight loss (10 lbs. in past year), self-reported exhaustion, weakness (grip strength), slow walking speed and low physical activity [7].

To preserve muscle strength, non-pharmacological interventions promoting physical exercise or improving nutrition could be important. For instance, physical exercise is reported to preserve muscle quality and strength, but also help prevent dependency [17, 18]. Furthermore, a nutritional approach with qualitative (e.g. sufficient protein intake) and quantitative (e.g. sufficient calorie intake) nutritional factors and a higher dietary intake macro and micronutrients such as antioxidants contributes to preserve physical performance and strength [19]. Several studies have found evidence of a synergy between adapted physical activities and dietary advice to prevent mobility impairment and decrease in functional status [17, 20–24].

The systematic detection of physical impairment to be able to intervene in the negative vicious circle towards dependency is an important public health goal. Screening and prevention are 2 of the core tasks of GPs, which are often the first medical contact with older patients. There is, however, no guideline or recommendation to screen the risk of mobility limitation in older adults in primary care [25]. Mobility assessment in clinical general practice should be performed using simple, easy, and quick tools. Several tools have been developed to detect loss of mobility; identifying functional complaints in patients with mobility difficulties has proven to be a valid screening tool for detecting subsequent loss of mobility [11, 26, 27].

The aim of this study is to assess the benefits of a multimodal intervention for older adults with complaints of reduced mobility, screened systematically in primary care. The main objective of this study will be to evaluate between the control and intervention groups change in mobility and muscular force at 6 and 12 months of follow-up, evaluate physical performance, nutritional status, quality of life, and autonomy.

## Methods

### Study design

**Fig. 1:**
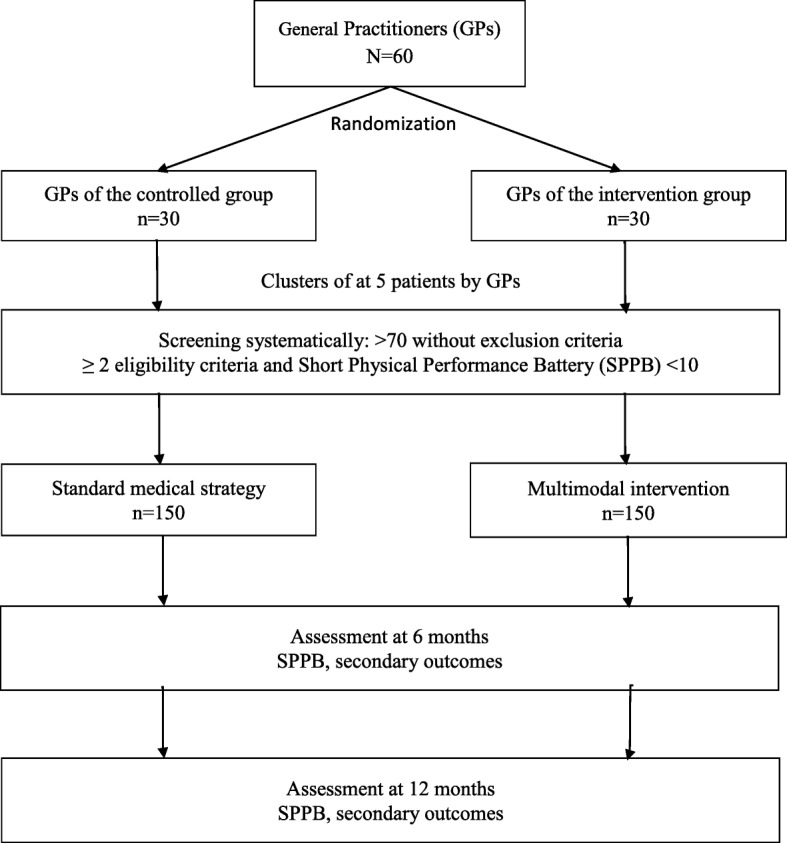
Design of the PRISME 3P randomized controlled trial

PRISME-3P is a national, multicenter, and cluster randomized trial conducted in France (Fig. 1). GPs will be randomly assigned to the intervention group or to the control group. Recruitment and inclusion will be performed by GPs and the intervention will take place at the hospital and home of the patient. The planned inclusion period is 24 months with 12 months follow-up.

In both groups, GPs will perform a consultation for inclusion at baseline and two evaluations at 6 and 12 months thereafter. In the intervention group, an initial blood test will be performed (blood cell count, CRP, pre-albuminemia, and albuminemia). Follow-up will include two visits to the allocated study hospital outpatient clinic (within 2 weeks and at 3 months from inclusion) and 3 visits to the GP (at 3, 6, and 12 months from inclusion). All data collected will be notified by the GPS on the case report form (CRF).

### Identification of areas of recruitment

First, we have searched for hospital that could participate in the study (having a geriatric unit with a dietician). We have invited 8 geriatric units to participate. Second, we have contacted the university general practice department of each hospital areas, i.e. Lyon (Lyon, Villefranche-sur-Saône, Bourg-en-Bresse, Annemasse), Nantes (Nantes), Saint-Etienne (Saint Etienne) and Nice (Menton). In each department a coordinator will be designated with responsibility to recruit GPs near the hospital.

### Recruitment and randomization of GPs

A total of 60 GPs will be recruited in 8 areas of France (6 in Nantes, 6 in Dijon, 19 in Lyon, 8 in Villefranche-sur-Saône, 5 in Bourg-en-Bresse, 5 in Annemasse, 6 in Saint-Etienne, and 5 in Menton). They will be assigned to the intervention group or to the control group using the survey select procedure of SAS® (SAS Institute Inc., Cary, NC, USA). The sampling will be stratified on the referring geriatric center (i.e. city). GPs working in the same practice will be considered as a unique sampling unit to avoid risk of contamination. Each collaborating university general practice department will organize training for the screening and assessment of patients required by the study protocol, including the provision of information on the eligibility criteria and study organization.

### Participants

All subjects aged over 70 years old will be eligible except patients with functional or locomotor disabilities, a life expectancy less than 6 months, a progressive chronic disease affecting participation in the program, or a MMSE< 20.

For each consecutive patient over 70 years of age without exclusion criteria, GPs will screen patients with medical interview to detect 2 or more inclusion criteria described in Table 1. These criteria are partly derived from the sarc-f screening tool for sarcopenia [28].

**Table 1:** Summary of eligibility, inclusion, non-inclusion and exclusion criteria

Eligibility criteria	Inclusion criteria	Exclusion criteria
Difficulty lifting packages over 4.5 Kg	Age > 70	Patient refusal to participate to the study
Difficulty rising from low seats without help of arms	SPPB <10	Functional or locomotor disabilities
Difficulty climbing less than 10 steps of stairs	≥ 2 eligibility criteria	Life expectancy less than 6 months
Difficulty moving or reduced walking speed		A chronic disease affecting participation to the program
Difficulty walking over 400 m without break		MMSE< 20
Walking time less than 1 hour per week		
Feeling of exhaustion during domestic activity		
> 2 falls during the last year		
Involuntary weight loss (> 5% in 1 month or > 10% in 6 months)		

In the presence of 2 or more inclusion criteria, GPs will assess the patient using the Short Physical Performance Battery (SPPB) [29]. In the event of a SPPB score < 10 (out of 12), patients will be given 9 questionnaires grouped in a unique folder: Activity Scale for the Elderly (PASE) [30], weekly sedentary time inspired by Gardiner [31], Activity of daily living scale (ADL), Instrumental activities of daily living scale (IADL) [32], SF-12 scale [33], Mini-Nutritional Assessment (MNA) [34], EPICES score *(Evaluation de la précarité et des inégalités de santé dans les centres de santé (in French))* [35, 36], Charlson index [37], Geriatric Depression Scale (GDS) [38] (Table 2) that measure their autonomy, nutrition and physical activity, and will be proposed another medical appointment for inclusion in the study. If a patient refuses to participate in the study, this will be noted in the CRF.

**Table 2:** List of questionnaires

List of questionnaires	
Physical activity	Activity Scale for the Elderly (PASE) [30]
	Weekly sedentary time inspired by Gardiner [31]
Autonomy	Activity of Daily Living scale (ADL)
	Instrumental Activities of Daily Living scale (IADL) [32]
Quality of life	SF-12 scale [33]
Nutrition	Mini-Nutritional Assessment (MNA) [34]
Patient’s social environment	EPICES score [35, 36]
Comorbidities	Charlson index [37]
Depression symptoms	Geriatric Depression Scale (GDS) [38]

### Multimodal and multidisciplinary intervention

**Fig. 2:**
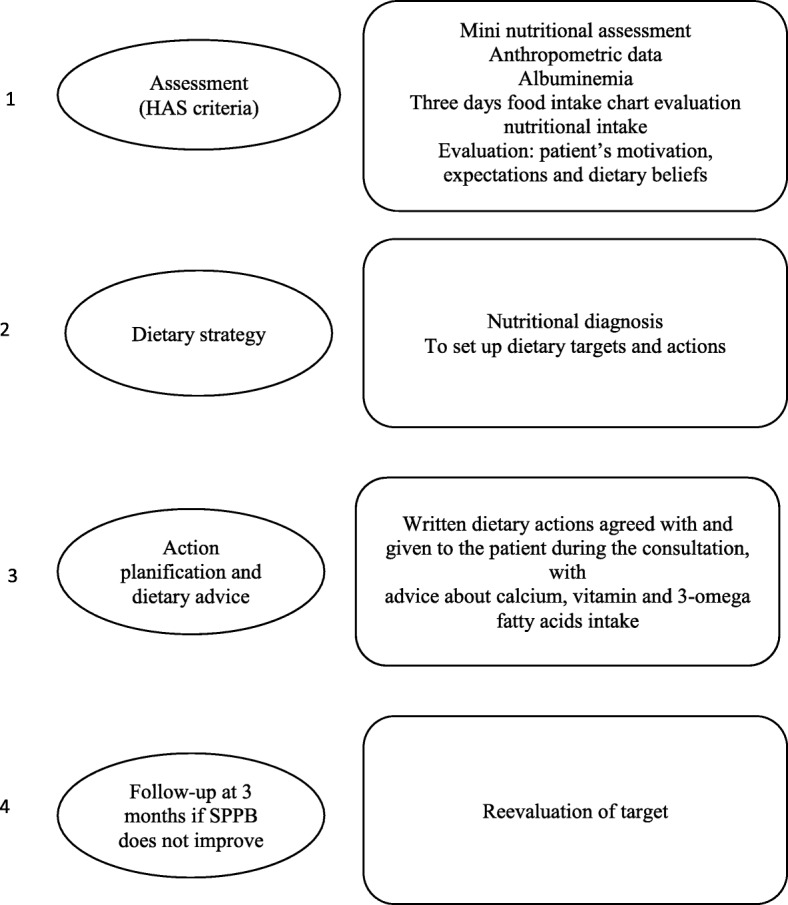
Nutritional intervention. It is a 3-step intervention with as assessment, an establishment of target and actions, and an action planning in collaboration with the patient

At the hospital, a three-step intervention are planned. First, participating geriatricians will perform a standardized geriatric assessment of each patient to rule out underlying diseases that might cause exhaustion or weight loss and hamper participation in the study. Second, a dietician from the study center will evaluate the nutritional status of patients (Mini Nutritional Assessment, weight, Body Mass Index, weight loss) and analyse the dietary intake reported on a three-day survey given to the patients to complete prior to the visit. On the day, the dietician will also perform calorimetric measurements to estimate dietary needs of each patient. A dietary strategy will be planned and agreed with the patients. The dietician will give written advice to each patient about calorie, protein, calcium, omega-3 fatty acid, and vitamin requirements (Fig. 2). Third, the patient will meet a personal trainer, who is specialized in physical exercise in the older age. A physical training program has been developed for the purpose of the study. It is based on existing validated training programs, with sets of exercises dedicated to three specific targets: muscle reinforcement, balance/flexibility, and endurance. It will consist of daily exercises lasting approximately 30 min and performed 5 days a week. These exercises are simple and require no specific equipment. Three sessions are dedicated to muscle reinforcement (lower limbs, upper limbs, and chest), and two to balance/flexibility and endurance. Intensity will increase over time to improve muscle strength, tailored to the abilities of individual patients. This program will be supported by a dedicated personal trainer, who will evaluate the patients: the personal trainer will explain and demonstrate each exercise to the patient (and caregiver), then the patient will practice each exercise in front of the monitor and be given the opportunity to rectify, and once the monitor has checked that the exercises are fully understood, he/she will hand the patient and caregiver a written handbook with illustrated explanations of the exercises for home training. Patients will be asked to report their activity and adherence to the program in the handbook. A monthly telephone follow-up will be set up, by which the monitor will be able to provide some advice and encouragements to the patients.

By contrast with the control group, patients in the intervention group will visit the GP at 3 months, who will perform an intermediate SPPB assessment in order to adapt the intensity of the intervention. If SPPB improves by one point or more, no change will be planned, if not they consult the same geriatrician and dietician, and will receive again the physical training program from the personal trainer who will demonstrate the exercises and propose individual (home) or group training sessions (hospital) twice a week for 10 weeks.

### Outcomes and measurements (Table 3)

#### Primary outcome

The primary outcome will be the difference in SPPB scores between inclusion and 6-month visits. To allow comparison, the SPPB will be evaluated by the same GP as far as possible. The SPPB measure reflects the performance status in 3 dimensions: balance, gait, strength/endurance are evaluated by examining ability to stand with the feet together in the side-by-side, semi-tandem and tandem positions, time to walk 4 or 6 m, and time to rise from a chair and return to the seated position 5 times [39].

**Table 3:** Schedule of outcome assessments

	Baseline	Intervention Group			Control Group	
		M 3	M 6	M 12	M 6	M 12
SPPB score	X	X	X	X	X	X
Walk test distance	X		X	X	X	X
SPPB	X	X	X	X	X	X
Physical activity	X		X	X	X	X
ADL/IADL	X		X	X	X	X
SF 12	X		X	X	X	X
Serious falls assessments	X		X	X	X	X
Hospitalization	X			X		X
Institutionalization				X		X
Weight, BMI	X	X	X	X	X	X
MNA	X					
Social score	X					
Charlson Index	X					
Medication inventory	X					

*PASE* Physical Activity Scale for the Elderly, *SF12* 12-Item Short Form Health Survey, *BMI* Body Mass Index, *MNA* Mini Nutritional Assessment, *CRP* C-Reactive Protein, *GDS* Geriatric Depression Scale, *GP* General Practitioners, *EPICES Evaluation de la précarité et des Inégalités de santé dans les centres de santé* (in French)

#### Secondary outcomes

The change of the secondary outcomes between the inclusion, 6, and 12-month follow-up will be analyzed. Secondary outcomes are: physical performance evaluated by the PASE [30] and sedentary behavior inspired by Gardiner et al. [31], and the distance walking; autonomy evaluated by the ADL and IADL [32]; quality of life evaluated by the SF-12 scale [33]; the number of serious injuries, hospitalizations, and institutionalizations, falls with healthcare consumption; nutritional status: weight, BMI, albuminemia (adjusted on CRP level) and MNA [34].

Additional outcomes will estimate prevalence of patients over 70 years of age with mobility limitation, adherence of patient and GPs to the program, and describe geriatric diagnosis after the consultation for the intervention group.

At baseline, GPs will collect data from the patients social environment EPICES score) comorbidities (Charlson index) [37], depression symptoms (GDS) [38], and medication inventory.

### Sample size calculation

The inclusion of 28 GPs per arm will provide a power of 80% to detect a difference of 0.8 points in SPPB score change between arms (1.2 points increase in the control group, versus 2 points in the intervention group), with 5 patients for each GP, an intra-cluster correlation coefficient of 0.15, and with a standard deviation of inter-individual variability of 1.7. Accounting for loss to follow-up, it is planned to randomize 30 GPs in each group, giving a total of 60 GPs and 300 patients (5 patients for each GP). The power of the study was calculated using the method described by Donner and Klar [40] and with the package CRTsize in the R program, version 3.3.1 (R foundation, Vienna, Austria).

### Statistical analyses

The patient characteristics at inclusion will be described according to each group (intervention and control groups). A summary table will be provided in order to ensure the homogeneity of the prognostic factor distributions according to the groups. A second summary table of clusters (GPs) will also be provided. The method and the presentation of the results will be according to the guidelines for cluster trials [41].

#### Primary outcome

All analyses of the primary outcome will be performed on the intent-to-treat population. The distribution of the primary outcome (difference in SPPB scores between inclusion and 6-month visits) will be described and presented for the two groups (graphical representation, e.g. boxplot). The primary outcome will be analyzed using a multiple linear regression model. When the conditions for application of the linear model are not respected (normality of residuals, independently distributed residuals…), a transformation (logarithmic or even Box-Cox, quantile, or z-score) of the SPPB score will be considered.

The main analysis of the primary outcome will be adjusted on the center using a mixed effect linear model with a random GP effect, and as fixed effects: an intervention effect (the control group as reference), a period effect (the inclusion as reference), and an interaction between treatment and period. This model will also be adjusted on the age at inclusion. An interaction between the SPPB at inclusion and intervention will be added to the model to test whether the treatment effect is different for patients with lower vs. higher level of SPPB score at inclusion. *P*-values and 95% confidence intervals of the fixed effects will be provided. Another analysis using the SPPB score as independent variable in a longitudinal mixed effect linear model will also be considered.

### Secondary outcomes

The analysis used for the primary outcome will be used to evaluate the change of the secondary outcomes between the inclusion and the 6-month follow-up visit.

The analysis of the change of secondary outcomes in the interventional group will be performed using a longitudinal mixed effect linear model with a fixed period effect and as random effects: the center, the GP, and the patient nested within the GP. This analysis will be adjusted on the age at inclusion.

A univariate analysis will be performed using a longitudinal mixed effect linear model to identify the factors associated with the SPPB result at 6 months in the interventional group. The model will include as random effects the center, the GP and the patient nested within the GP, and as fixed effect the corresponding factor. This analysis will provide the effect estimation of each factor with its 95% confidence intervals. The independent factors found significant in this univariate analysis will be included in a multivariate analysis.

All analysis will be performed using the statistical analysis program SAS® version 9.4 (SAS Institute Inc., Cary, NC, USA), and the R program, version 3.3.1 (R foundation, Vienna, Austria).

### Ethics approval and consent to participate

The study protocol was approved by the Sud Est 4 Ethics Committee on October 18, 2016 and cover all sites involved in this study. The research carried out will be on accordance with the Helsinki Declaration and ICH GCP Guidelines. The study complies with the principles of the data protection act in France. Each GP had to collect a writer consent at the beginning of the procedure. This consent is retained in the CRF. The patient can stop the study at any time with an oral information at his GP.

## Discussion

The majority of studies are based on non-pharmacological interventions to prevent mobility impairment. This large-scale randomized trial is a program for older patients screened in primary care. To the best of our knowledge, this will be the first study in France in which a multimodal intervention with cooperation between geriatricians, general practitioners, dieticians, and personal trainers will be implemented.

### Discussion of the study design

The study will be randomized and multicenter, and will investigate the effect of a specific clinical pathway with both the effects of the intervention and, simultaneously, the implementation of the program in current practice conditions, across various areas of France. Rather than an individual randomization, a cluster design was chosen in order to limit the risk of contamination, but also for reasons of feasibility and ethics; it would have been difficult to justify why two different patients in the same practice would not have access to the same management. Likewise, we chose to stratify randomization to ensure that GPs working in the same practice would be randomized to the same group. The high number of clusters (60 in total) and low number of patients within each cluster should help reduce the risk of bias due to intra-cluster correlation.

Despite the cluster design, the effects of the intervention will be determined individually. An open-label design is necessary given the nature of the intervention. The effects of the intervention will be evaluated using an objective outcome, the SPPB, which has shown to be reproducible [29, 39]. An SPPB score below 10 is predictive of all-cause mortality [42] and reveals a weakness of lower extremity function and higher mobility disabilities [43]. Clinically, an improvement of one point in the SPPB score is associated with substantial and clinically relevant changes in physical performance [44]. SPPB is a reliable tool to screen for patients likely to benefit from a physical exercise program [41] and it is generally considered as a valid predictive tool to assess physical performance and predict risk of mobility loss [11, 39, 42]. The GPs will screen systematically each consecutive patient over 70 years of age to limit selection bias. Furthermore, they will note each refusal to participate in order to evaluate the acceptability of the program from the patients’ perspective.

Standard training will be provided to the participating GPs for the recruitment and to physical trainer to ensure harmonized delivery of information. All GPs will be given specific training in group sessions for the loss of mobility screening assessment, prior to randomization. This will ensure that the screening and inclusion procedure is the same for all patients, and ensure comparability between groups for the measurement of the primary outcome. Moreover, GPs in the intervention group will not be involved directly in the intervention itself.

### Feasibility

Patients with an estimated life expectancy of less than 6 months will be excluded as they would derive no benefit from intervention. In France, patients choose their GP and generally trust him/her. We believe this privileged relationship might help acceptance and adherence to the program, and also limit the rate of patients lost to follow-up. Conversely, part of the intervention will take place in a hospital setting and this might limit the acceptability of the program. This was decided for feasibility reasons, as this limited the number of personal trainers required and helped to optimize their time by avoiding multiple home visits.

The recruitment of patients from primary care will be facilitated by the collaboration and institutional support of the university general practice departments in each participating area. To facilitate GP adherence and acceptability of a screening, GP needs a valid, simple and rapidly administered tool. The FRASI-Study found that the use of SPPB is feasible in daily practice in primary care [45]; it is a simple tool that has good performance to predict incident ADL disability, worsening mobility, and death [39, 43, 46]. We have estimated the time required to deliver the SPPB to be less than 20 min, which corresponds to the mean duration of a regular GP consultation in France. To minimize the duration of the inclusion visit, GPs will screen and assess patients and give them a questionnaire during a routine visit. They will be invited to read this questionnaire and answer the questions to the best of their abilities, which will be then completed with the help from GPs during a dedicated inclusion visit.

To the best of our knowledge, this will be the first study in France in which a multimodal intervention with cooperation between geriatricians, general practitioners, dieticians, and personal trainers will be implemented. We will be able to evaluate the implementation of a collaborative program that may help setting-up effective pathways for prevention of loss of mobility in primary care.

## Abbreviations

ADL: Activity Daily Life
BMI: Body Mass Index
EPICES: *Evaluation de la Précarité et des Inégalités de santé dans les Centres d’Examens de Santé* (in French)
GDS: Geriatric Depression Scale
GP: General Practitioner
IADL: Instrumental Activity Daily Life
MAPA: Monitor of Adapted Physical Activities
MMSE: Mini Mental State Examination
MNA: Mini Nutritional Assessment
PASE: Physical Activity Scale for the Elderly
PRISME-3P: Elderly Patient at RISk of loss of Mobility, Exercise – Primary care, Prevention, care Pathways
SPPB: Short Physical Performance Battery
